# Bedsores Management: Efficiency Simulation of a New Mattress Design

**DOI:** 10.3390/healthcare9121701

**Published:** 2021-12-08

**Authors:** Abdullatif Alwasel, Bandar Alossimi, Maha Alsadun, Khalid Alhussaini

**Affiliations:** 1 Department of Biomedical Technology, College of Applied Medical Sciences, King Saud University, P.O. Box 10219, Riyadh 11433, Saudi Arabia; Bandrossimi@gmail.com (B.A.); kalhussaini@ksu.edu.sa (K.A.); 2 Smart Innovation Inc., Riyadh 12221, Saudi Arabia; m-alsadun@hotmail.com

**Keywords:** bedsores, pressure ulcers, immobility

## Abstract

Bedsores, also known as pressure ulcers, are wounds caused by the applied external force (pressure) on body segments, thereby preventing blood supply from delivering the required elements to the skin tissue. Missing elements hinder the skin’s ability to maintain its health. It poses a significant threat to patients that have limited mobility. A new patented mattress design and alternative suggested designs aimed to reduce pressure are investigated in this paper for their performance in decreasing pressure. A simulation using Ansys finite element analysis (FEA) is carried out for comparison. Three-dimensional models are designed and tested in the simulation for a mattress and human anthropometric segments (Torso and Hip). All designs are carried out in solidworks. Results show that the original design can redistribute the pressure and decrease it up to 17% less than the normal mattress. The original design shows better ability to decrease the absolute amount of pressure on the body. However, increasing the surface area of the movable parts results in less pressure applied to the body parts. Thus, this work suggests changing the surface area of the cubes from 25 to 100 cm^2^.

## 1. Introduction

Bedsores, also known as pressure ulcers, are wounds that pose a significant threat to the wellbeing of limited mobility patients by the pressure influence on normal blood circulation. The pressure reduces the amount of oxygenated blood cells loaded with nutrients to the skin surface under pressure [1]. Data show that 70% of bedsores patients are 65 years old and older [1]. Moreover, the younger population with a severe or chronic illness accompanied by neurological impairment are more prone to developing bedsores due to the lack of movement. Bedsores are classified according to their severity into four stages starting with stage I where skin is intact with non-blanching redness [2,3,4]. Stage II is when the skin starts to open as an ulcer; stage III is when the skin starts losing full thickness tissue with visible subcutaneous. The last stage, Stage IV, is when there is a full-thickness skin loss with visible muscle and bone.

In the United States, bedsores patients’ prevalence was 32 cases/1000 patients in the period between 2005–2007, costing the healthcare system more than $2.4 billion U.S dollars [4]. These statistics indicate the high prevalence associated with the excessive cost of managing pressure ulcer wounds. Pressure ulcer wound management is essential, especially since the National Quality Forum [5] announced it as a preventable hospital-acquired illness. Institutions such as the Center for care and Medicaid no longer cover the costs of treating hospital-acquired illnesses [4]. This fact dictates that hospitals must focus on methods to prevent the development of pressure ulcers to reduce hospitalization time and the cost associated with it.

Ways to manage the development of pressure ulcers are not new to healthcare providers. Risk factors for developing pressure ulcers are divided into intrinsic and extrinsic [6]. While intrinsic factors are hard to manage mechanically due to the fact they lie within the body, extrinsic factors are often dealt with mechanically. There are multiple ways in which health practitioners can screen patients for pressure ulcers; since the early 1960s, risk assessment scales have been developed and used [7]. Among the most famous scales are Braden and Norton scales. These scales are 5 and 4-point scales to screen patients in order to prevent pressure ulcers from developing. The Braden and Norten scales are methods that medical practitioners use to get a prediction whether a patient is at risk of developing pressure ulcers and not ways to prevent the development. The Braden scale takes into account the sensory perception, activity, mobility, moisture, nutritional status, and shear. Furthermore, the Norton scale takes into account the general physical condition of patients, and their mental state, activity, mobility, and incontinence. According to the wound healing society [8], the process starts with the Pressure Ulcer Risk Screening (PURS) where all patients must undergo an assessment by a registered nurse to identify whether they are at risk of developing pressure ulcers. If identified, patients undergo a plan for prevention that includes dressing and pressure relief. Guidelines recommend three stages for the prevention of bedsore among patients, including screening where all patients with known risk factors such as age, sex, race are vetted for risk factors. Risk assessment is used to assess how likely the patient would develop the bedsores. Finally, wound management is used at a later stage where the ulcer has already been developed.

Bedsore wound management and prevention has been under the scope of researchers and scientists; due to this fact, multiple solutions have been developed. Mechanical or pneumatic tools emerged to aid healthcare practitioners such as mattresses and overlays that are divided into different design cells filled with gas or water [9,10] cushions designed on anatomical bases [11] and wedges to support limbs [12]; or a combined dynamic system of electrical sensors and mechanical components [13,14]. According to Marchione et al. 2015, the most used techniques to prevent and manage pressure ulcers are by monitoring pressure. Although many other techniques monitor blood flow, pressure distribution remains the main focus when it comes to pressure ulcer risk factor monitoring [14].

Given that the extrinsic risk factors of developing pressure ulcers are pressure, friction, and shear, the effect of these risk factors on the skin can be recreated using simulation techniques such as finite element analysis (FEA). The use of such a technique provides researchers with the ability to apply multiple scenarios without the chance of harming human participants, while at the same time, providing relatively high accuracy results that can be reliably considered. Simulation studies have been previously used in many biomedical studies [15,16,17].

A new patented mattress [18] was designed to minimize the chance of developing bedsores among bed-ridden patients. The proposed design offers a cheap and effective solution to manage bedsores in prone patients. This study aims to investigate whether the proposed new design is able to effectively reduce the risk of developing bedsores. The study is divided into two parts; first, studying the pressure distribution using multiple simulation scenarios namely full support (with no pressure distribution) and with supported pressure distribution (removing some support elements). Second is studying the effectiveness of slight design modification on these scenarios.

## 2. Materials and Methods

### 2.1. Mattress Design Model Preparing 

**Figure 1:**
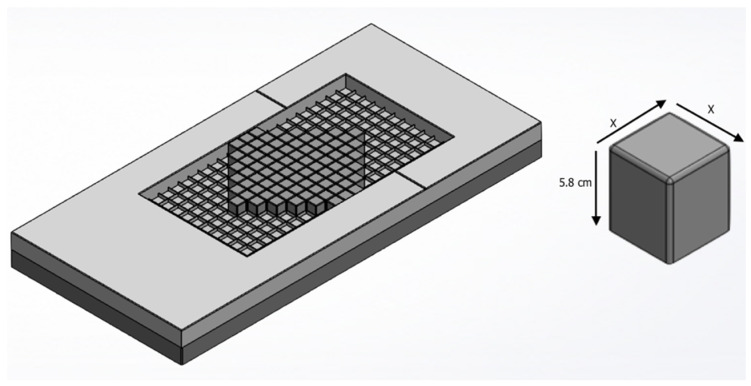
(**left**) Mattress Model Isometric view. (**right**) Isometric view of the cube model with its dimensions.

The mattress design three-dimensional drawing in Figure 1 is based on the patent measurements [18] developed using Solidworks^TM^ 2013. The patent design has two major components, one is the main mattress that is similar to any typical mattress, design-wise, with a width of 90 cm, length of 190 cm, and depth of (14 cm), and a cavity that spans the width of 50 cm, length of 110 cm, and depth of (7 cm), as shown in Table 1. The second component of the patented design is the removal cubes that are 5 × 5 × 5.8 cm.

The movable parts (cubes) mount in the mattress’s cavity to act as pressure-relieving points that can be extracted from underneath the patient’s body to alleviate and control the contact pressure on the body area supported. The cubes are placed and removed manually as needed. Once installed in their respective places, the cubes remain fixed statically by the internal grid.

The mattress cavity has a supporting grid shown in Figure 1 that holds every cube in place; the grid has a height of 2 cm with a thickness of 0.5 cm. Removing the cubes will be a solution to the static cushion deficiency in reliving pressure on bony landmarks [19]. The cubes can be removed, rearranged, and kept without alterations as a normal bed mattress according to the situation and the patient’s need. In this paper, the cubes that experience the maximum pressure during full support are removed to release and redistribute pressure.

Three different cube designs are tested in this paper, the original cube dimensions as per the patent, being the fundamental design. Moreover, two alternative designs were tested for lowering the contact pressure magnitude. Specifically, two different cube variations were introduced (Cube A and B) where only the width and length were changed. The different design measurement are shown in Table 1. All of the design models are carried out of Solidworks to form a 3D test subject.

### 2.2. Human Body Models 

**Figure 2:**
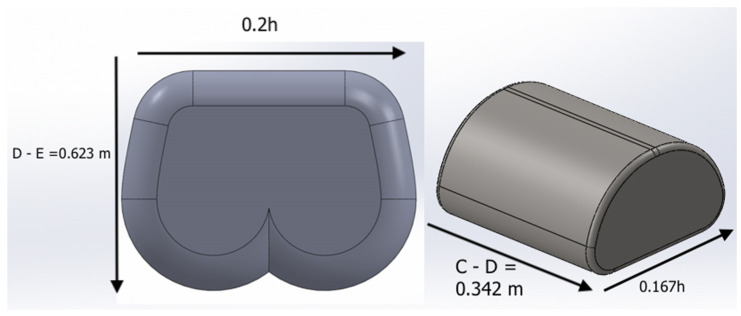
(**left**) Hip model bottom view. (**right**) Isometric view of torso model. Dimensions adapted from Huston anthropometric data.

The human body anthropometric models, shown in Figure 2, are designed to be the test tool placed on the mattress. The models’ measurements were adapted from Ronald L Huston [20] human anthropometric data. The area of interest in this paper is the upper body separated into two segments, namely torso and hip. These parts have the biggest surface area, providing the test with a clear contact pressure to form a result heat map on the models. The two segments are designed to assemble the upper body as torso and hip, as shown in Figure 1.

The 50th percentile of the normal distribution of the Huston anthropometric data [20] was used to design the body segments under test scope. The dimensions of the anthropometric models were obtained relative to height (h), as shown in Figure 2. The average height was set to 175.9 cm, torso height was set to 34.2 cm, and the hip height equals 26.3 cm. The corresponding weights based on an average body weight of 80.42 kg: torso weight was set to 29.53 kg, and the hip weight was set to 10 kg. All the parameters of the models were calculated using Ralph Huston principals of biomechanics [20].

The designs were exported after finalizing them using Solidworks^TM^ 2013 to simulate how contact pressure acts before and after distribution using ANSYS^TM^ release R 15.0.

### 2.3. Study Design and Simulation 

Instead of using pressure sensitive sensors [21], the simulation in this study was conducted Using ANSYS workbench finite element analysis to evaluate the contact pressure risk factor. To simulate a real case scenario, the analysis is set with the following boundary conditions:

1. Gravitational acceleration (g) = 9.81 m/s^2^.
2. Fixed support under the mattress.
3. Unstructured triangular mesh.
4. Minimum mesh size = 1 mm
5. Maximum mesh size = 1 cm

These conditions are selected to investigate the contact pressure exerted on the anthropometric model segments while the patient is in the supine position assuming the patient is immobile. The aim of this simulation is to draw a map of contact pressure distributed on the human body anthropometric models and to measure the mattress capability in reducing the contact pressure.

The study design was divided into two scenarios to compare the contact pressure resultants. Scenario I was set with a mattress containing full cubes to act as a normal mattress to determine the first comparison result, which is the highest contact pressure value on both the torso and hip. Scenario II was set after removing the cubes that support the highest contact pressure points on the model’s segment. Then, results from both scenarios were compared for design validation.

The pressure results on the model’s surface that are in contact with the mattress are inspected with ANSYS^TM^ Maximum principle stress and Von-Mises evaluation tool; furthermore, the same tool was applied to the whole mattress and anthropometric models to evaluate the behavior and response of the whole test objects. Then, the maximum principle pressure is used to carefully adjust and redistribute the cubes to relieve the high contact pressure points on the anthropometric models.

The Von-Mises provide results in three streams:

1. The pressure distribution on the whole system (inclusive of body part and mattress). This allows for investigating how the movable parts behave under pressure.
2. The pressure distribution on the torso model independently. This allows for the investigation of the effect of cube removal on the torso.
3. The pressure distribution on the hip model independently, which allows for investigating what happens to the hip when the arrangement of the cubes is changed.

Thus, the Von-Mises tool is capable of testing whether the different variation and the way they move affect the pressure distribution, which is sufficient for the purpose of this study.

All cube parts are designed similar to the original cube design with the depth left without changes. The tested cube dimensions are:

1. The original cube design: 5 × 5 × 5.8 cm
2. First cube design variation: 7 × 7 × 5.8 cm
3. Second cube design variation: 10 × 10 × 5.8 cm

The material chosen for the mattress and cubes is polyurethane foam. It is the typical material for the medical mattresses. The properties are:

- Density: 0.00800–4.5 g/cc
- Modulus of elasticity: 0.000138–3.45 GPa
- Tensile strength: 0.0207–1900 MPa
- Poisson’s ration: 0.30–0.75

## 3. Results

All results from the simulated force (bodyweight) are presented in Table 2. Full support refers to the mattress while all parts are intact. Supported pressure distribution refers to the mattress after the removal of some movable parts (the parts supporting the maximum pressure point). The number of parts removed differs for each body segment and ranges from one to five parts.

### 3.1. Original Cube Design 

The design cube dimensions are 5 × 5 × 5.8 cm, producing a total of 231 movable parts. The maximum local pressure, while the body is fully supported (complete parts), was ≃50 kPa on the Hip segment. Moreover, the maximum local pressure after pressure release (pressure distributed) was ≃41 kPa.

Furthermore, the maximum pressure on the Torso segment, while fully supported, was ≃51 kPa. After pressure release, the maximum pressure on the Torso was reduced to ≃44 kPa. The number of parts was reduced to 225 to obtain this pressure reduction on the hip.

### 3.2. Cube A Design 

Cube A dimensions are 7 × 7 × 5.8 cm with a total of 112 movable parts, which resulted in a maximum local pressure when the body was fully supported (complete parts), with ≃68 kPa on the hip segment. Moreover, the maximum local pressure after pressure release (pressure distributed) was ≃67.5 kPa.

Moreover, the maximum pressure on the torso segment, while fully supported, was ≃71 kPa. After pressure release, the maximum pressure on the torso registered a value of ≃69 kPa. The number of parts was reduced to 109 to obtain this pressure reduction. 

### 3.3. Cube B Design 

Cube B dimensions are the largest of the three designs and set to 10 × 10 × 5.8 cm; they generated a total of 66 movable parts, which lowered the maximum local pressure compared to the first two designs. While the body was fully supported (complete parts), the maximum local pressure was ≃25 kPa on the hip segment. Furthermore, the maximum local pressure after pressure release (pressure distributed) was ≃22 kPa.

Moreover, the maximum pressure on the torso segment, while fully supported, generated ≃41 kPa. After pressure release, the maximum pressure on the torso was reduced to ≃35 kPa. The number of parts was reduced to 57 to obtain this pressure reduction.

## 4. Discussion

This paper presents a simulation of consistent application of force (bodyweight) on a semi-rigid surface (mattress). The simulation mimics the case of a bed-ridden patient who is unable to move his/her upper body parts, and thus, is prone to bedsores.

The original patented design, shown in Figure 1, performed well as the original design. It was able to decrease the maximum pressure by 17% and 12% on the hip and torso consequently. These values show better performance compared to Cube A design that showed only a 0.7% and 1.71% on the hip and torso consecutively. Cube B design also showed a reduction of 10% and 14% for the hip and torso consecutively, as seen in Table 3.

Initially, before the modification of the arrangement of the movable parts and from results listed in Table 2, the pattern of pressure distribution follows:

P = F/A(1)

where P is the pressure, F is the applied force, and A is the surface area. Thus, as the surface area increases, less pressure is applied on each cube.

Moreover, the data shows an inverse relationship between the size and percentage of pressure release where, as we increase the movable part dimensions, the percentage of pressure change decreases on the hip part and almost stays constant on the torso. This should conclude that a larger dimension shows a better design.

However, the evaluation of the design falls into two categories, one is the pressure distribution performance, the other is the cost of production of the mattress, hence its affordability for home and clinical use. First, regarding the pressure distribution performance, initially before any modification it is clear that as the surface area increases, the performance is better as Cube B design with 10 × 10 × 5.8 cm shows a maximum of 25 and 41 kPa for the hip and torso consecutively before modification compared to a maximum of 50 and 51 kPa for the 5 × 5 × 5.8 cm. This suggests that increasing the surface area from 25 to 100 cm^2^ lowers the pressure by 50% and 35% for the Hip and Torso consecutively.

**Figure 3:**
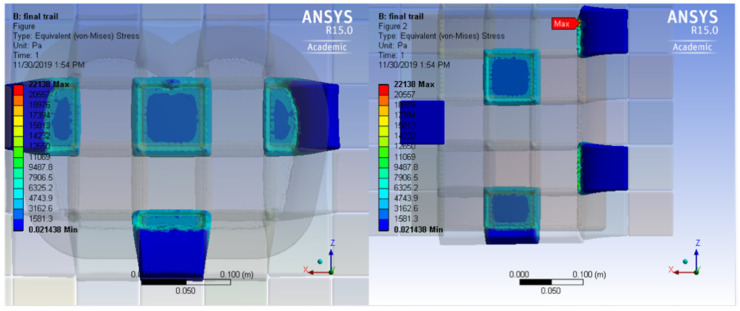
Pressure distribution within cube designs shows increased pressure on cube edges compared to center.

Investigating the pressure distribution within each cube shows that the pressure is concentrated on the edges of each cube, as shown in Figure 3, and the lowest values occur in the middle. This undesired behavior can be attributed to the nature of the material used to design the mattress (Polyurethane foam). This material is selected for its medical uses, however, it allows for horizontal expansion parallel to the top view of the cube. This behavior causes shear pressure from one cube to the adjacent cubem raising the amount of pressure on the cube as we decrease the surface area.

As a result, the pressure values do not decrease significantly by modification of cubes, as we increase the surface area from 25 to 49 and 100 cm^2^.

Although, the original design shows a better percentage of pressure distribution, the overall pressure, after distribution, is higher than the value before distribution, as shown in Table 2, for Cube A and B designs. These results follow the same initial line of thought that states, as we increase the surface area, the pressure applied at the cube is decreased. Thus, it can be concluded that from a performance point of view, it is advised to select the Cube B design for its ability to decrease the initial pressure at the start. However, this comes at a tradeoff between the amount of initial pressure and the ability to circulate pressure using movable parts modification.

As the aim of creating the mattress, under testing in this paper, is to decrease the likelihood to develop pressure ulcers among bedridden patients, the ability to circulate pressure is an essential element. Although the pressure is distributed better using the larger design, a patient-specific classification scheme must be created to allow for better results based on a case-by-case scenario. This is because the initial pressure distribution is a value that is directly proportional to the subject weight such that as the patient weight is increased the amount of pressure increases accordingly.

Furthermore, the current design is based on the cubical shape that was chosen to ease the process of modifying the mattress arrangement, as needed, for the purpose of pressure circulation. It is similar to previously published work where a pneumatic system is used to create patterns of pressure support points [10]. However, although the presented design is cheaper to manufacture, a major drawback of this shape is although the current design shape provides good results in lowering pressure, the contact area between one cube and the other forms an area of pressure concentration, as shown in Figure 3. Although this pressure concentration might not be a primary source of pressure ulcer, it remains a concern.

The results of this study were produced under specific conditions, and thus, its results cannot be generalized to the overall population. It is important to note that the scope of work in this paper is the design variation and its ability to reduce pressure without considering gender and/or population percentile. Further human participant testing must be carried out to be able to generalize results.

## 5. Conclusions

A new mattress design is investigated for the use of bedsores management among bedridden patients. The design was recently granted a patent from the Saudi patenting office [18]. Consisting of two parts, this mattress uses the concept of lean design to enable pressure distribution, and hence, blood circulation and supply to prone body areas.

This paper uses FEA to simulate the effect of upper body weight on the mattress in three scenarios with varying designs. The aim is to investigate whether the proposed design variation is able to effectively reduce the risk of developing bedsores. The original design shows better ability to decrease the absolute amount of pressure on the body. However, increasing the surface area of the movable parts shows that less pressure is applied to the body parts.

In conclusion, although the original design allows for decreasing the amount of pressure by rearranging the parts, the higher the surface area, the lower the pressure. Thus, this work suggests changing the surface area of the cubes from 25 to 100 cm^2^. Further research is required to investigate whether alternative movable parts’ shapes can enhance the performance of pressure distribution around the edges of the movable parts. 

## Figures and Tables

**Table 1:** 3D Models measurements, the two parts (Frame and cavity) of the mattress and the original cube are taken from the patent. Cube A and B are introduced as alternatives.

Object		Length cm	Width cm	Depth cm
Mattress	Frame	190	90	14
	Cavity	110	50	7
Original cube		5	5	5.8
Cube A		7	7	5.8
Cube B		10	10	5.8

**Table 2:** Pressure distribution resulting from simulating force application on three different mattress designs.

Pressure	Maximum Local Pressure (pa)			
	Design Variations	Segment	Pressure	Cubes Removed
Full support	5 × 5 × 5.8 cm	Hip	50,167	0
		Torso	51,151	0
Supported pressure distribution	5 × 5 × 5.8 cm	Hip	41,613	5
		Torso	44,952	1
Full support	7 × 7 × 5.8 cm	Hip	67,997	0
		Torso	71,002	0
Supported pressure distribution	7 × 7 × 5.8 cm	Hip	67,520	1
		Torso	69,789	2
Full support	10 × 10 × 5.8 cm	Hip	25,414	0
		Torso	41,303	0
Supported pressure distribution	10 × 10 × 5.8 cm	Hip	22,830	5
		Torso	35,352	4

**Table 3:** Percentage of pressure change obtained from simulating the force application on three different cube designs.

Segment	Pressure Comparison	
	Design Variation Set	Pressure Change Total Percentage
Hip	5 × 5 × 5.8 cm	17.05%
Torso		12.12%
Hip	7 × 7 × 5.8 cm	0.70%
Torso		1.71%
Hip	10 × 10 × 5.8 cm	10.17%
Torso		14.41%

## Data Availability

The data presented in this study are available on request from the corresponding author.
